# Supplementary material for: Dietary Fibres and the Management of Obesity and Metabolic Syndrome: The RESOLVE Study
Source: Nutrients. 2020 Sep 23;12(10):2911. doi: 10.3390/nu12102911 (PMC7650763; doi:10.3390/nu12102911)
Supplement: Supplementary file 1 [file nutrients-12-02911-s001.zip › supplementary materials/2020-09-22_TableS1_EffectOfNutritionalIntakeOnHealthOutcomes.docx]

**Table S1.** Effect of nutritional factors on health outcomes - Effect size (95% confidence intervals); p-value.

Data adjusted on age (data not shown). Significant effect sizes are in bold. This table is the detail of Figure 2. HbA1c: glycated haemoglobin ; Homa-IR: Homeostatic Model Assessment of Insulin Resistance, HDL: high-density lipoprotein, LDL: low-density lipoprotein, Hs-CRP: high-sensitivity c-reactive protein. BMI: Body Mass Index. SBP: systolic blood pressure; DBP: diastolic blood pressure. Re, rE and re: see methods for details. D: day; M: month.

|  |  |  |  |  |  |  |
| --- | --- | --- | --- | --- | --- | --- |
|  | **Central fat** | **Weight (x1000)** | **BMI (x1000)** | **Fat mass** | **Fat free mas** | **Waist (x1000)** |
|  |  |  |  |  |  |  |
| **Energy intake (kcal/day)** | 0.26 (-0.12 to 0.64); p=.184 | 3.49 (-0.82 to 7.80); p=.113 | 0.50 (-1.15 to 2.15); p=.553 | 1.15 (-2.06 to 4.37); p=.483 | **1.69 (0.17 to 3.21); p=.030** | **6.10 (1.16 to 11.0); p=.016** |
| **Protein intake (g/day)** | -0.96 (-5.40 to 3.49); p=.674 | -3.63 (-53.5 to 46.2); p=.887 | 4.52 (-14.6 to 23.7); p=.643 | 1.20 (-36.0 to 38.4); p=.949 | 3.40 (-13.1 to 19.9); p=.687 | 2.94 (-53.7 to 59.6); p=.919 |
| **Carbohydrate intake** |  |  |  |  |  |  |
| Complex carbohydrate (g/day) | **2.28 (0.37 to 4.17); p=.019** | 14.5 (-6.78 to 35.8); p=.182 | 7.24 (-0.94 to 15.4); p=.083 | 13.5 (-2.44 to 29.4); p=.097 | 2.92 (-4.42 to 10.3); p=.436 | 8.51 (-15.9 to 32.9); p=.494 |
| Simple carbohydrate (g/day) | 0.96 (-2.26 to 4.18); p=.560 | 14.8 (-21.7 to 51.3); p=.427 | 7.02 (-6.95 to 21.0); p=.324 | 17.0 (-10.1 to 44.2); p=.219 | -6.58 (-19.0 to 5.79); p=.297 | 16.6 (-24.5 to 57.7); p=.429 |
| **Lipid intake** |  |  |  |  |  |  |
| Saturated fat (g/day) | -9.16 (-19.6 to 1.28); p=.086 | -111.6 (-228.7 to 5.5); p=.062 | -24.6 (-69.6 to 20.4); p=.284 | -58.2 (-145.8 to 29.3); p=.192 | -7.77 (-50.4 to 34.8); p=.721 | **-149.1 (-282.8 to -15.3); p=.029** |
| Monounsaturated fat (g/day) | -3.40 (-16.9 to 9.80); p=.600 | 210.0 (-19.5 to 440.1); p=.073 | -23.6 (-99.3 to 52.0); p=.540 | **-148.7 (-293.8 to -3.7); p=.044** | **-50.5 (-93.7 to -7.3); p=.022** | 123.6 (-41.0 to 288.2); p=.141 |
| Polyunsaturated fat (g/day) | 7.2 (-7.2 to 21.4); p=.330 | 98.2 (-62.1 to 258.5); p=.230 | 25.1 (-36.5 to 86.7); p=.425 | 100.7 (-19.3 to 220.7); p=.100 | -7.7 (-62.3 to 47.4); p=.791 | 37.6 (-146.5 to 221.6); p=.689 |
| Cholesterol intake (mg/day) | **1.39 (0.76 to 2.02);** **p<.001** | **15.1 (8.06 to 22.1); p<.001** | **5.36 (2.66 to 8.07); p<.001** | **10.7 (5.46 to 16.0); p<.001** | **4.61 (2.28 to 6.94); p<.001** | **15.9 (7.80 to 24.0); p<.001** |
| **Fibre intake (g/day)** | **-19.1 (-28.2 to -10.0); p<.001** | **-202.1 (-304 to -99.9); p<.001** | **-73.6 (-113 to -34.4); p<.001** | **-130 (-207 to -54.3); p<.001** | **-48.1 (-82.7 to -15.1); p=.005** | **-275 (-392 to -158); p<.001** |
| **Water intake (ml/day)** | 0.02 (-0.07 to 0.10); p=.735 | -0.39 (-1.39 to 0.60); p=.437 | -0.02 (-0.40 to 0.37); p=.932 | -0.08 (-0.83 to 0.66); p=.827 | -0.12 (-0.45 to 0.21); p=.471 | 0.19 (-0.92 to 1.30); p=.744 |

|  |  |  |  |  |  |  |
| --- | --- | --- | --- | --- | --- | --- |
|  | **Blood glucose (x1000)** | **HbA1c (x1000)** | **Insulimenia** | **Homa-IR (x1000)** | **Total cholesterol (x1000)** | **Triglycerides (x1000)** |
|  |  |  |  |  |  |  |
| **Energy intake (kcal/day)** | 0.28 (-0.48 to 1.03); p=.472 | 0.10 (-0.27 to 0.48); p=.597 | **-1.53 (-2.89 to -0.17); p=.027** | -0.96 (-2.45 to 0.52); p=.206 | -0.28 (-0.96 to 0.40); p=.419 | 0.51 (-0.01 to 1.03); p=.055 |
| **Protein intake (g/day)** | 5.0 (-3.7 to 13.7); p=.260 | 0.10 (-4.22 to 4.43); p=.963 | **23.6 (7.56 to 39.6); p=.004** | **26.0 (8.58 to 43.3); p=.003** | **-10.0 (-17.9 to -2.18); p=.012** | **-8.67 (-14.4 to -2.34); p=.007** |
| **Carbohydrate intake** |  |  |  |  |  |  |
| Complex carbohydrate (g/day) | **4.86 (1.11 to 8.60); p=.011** | **1.88 (0.02 to 3.74); p=.048** | **8.76 (1.84 to 15.7); p=.013** | **9.76 (2.26 to 17.3); p=.011** | **6.65 (3.28 to 10.0); p<.001** | **2.67 (0.08 to 5.27); p=.043** |
| Simple carbohydrate (g/day) | -3.49 (-9.62 to 2.64); p=.264 | -2.48 (-5.60 to 0.64); p=.119 | **11.8 (1.11 to 22.5); p=.031** | 5.93 (-5.97 to 17.8); p=.329 | 0.72 (-4.97 to 6.30); p=.801 | 0.16 (-4.09 to 4.4); p=0.942 |
| **Lipid intake** |  |  |  |  |  |  |
| Saturated fat (g/day) | -11.9 (-32.3 to 8.59); p=.256 | -4.39 (-14.6 to 5.79); p=.398 | -0.06 (-37.4 to 37.3); p=.998 | -13.8 (-54.5 to 26.9); p=.506 | **23.2 (4.81 to 41.7); p=.013** | -1.99 (-16.2 to 12.2); p=.783 |
| Monounsaturated fat (g/day) | 4.79 (-10.9 to 20.5); p=.549 | 2.69 (-8.33 to 13.71); p=.632 | 7.68 (-11.7 to 27.1); p=.437 | 11.9 (-12.4 to 36.2); p=.338 | -4.3 (-20.5 to 12.0); p=.605 | -1.90 (-12.7 to 8.89); p=.730 |
| Polyunsaturated fat (g/day) | 23.7 (-4.61 to 51.9); p=.101 | 9.90 (-4.13 to 23.9); p=.167 | 28.9 (-23.3 to 81.2); p=.277 | 48.5 (-8.12 to 105.1); p=.093 | -6.07 (-31.5 to 19.4); p=.640 | -12.3 (-31.9 to 7.33); p=.220 |
| Cholesterol intake (mg/day) | 0.93 (-0.30 to 2.17); p=.139 | **0.68 (0.07 to 1.30); p=.029** | **-2.29 (-4.59 to -0.00); p=.050** | -1.67 (-4.14 to 0.81); p=.188 | -1.09 (-2.20 to 0.03); p=.056 | -0.14 (-0.99 to 0.72); p=.755 |
| **Fibre intake (g/day)** | **-34.2 (-52.1 to -16.3); p<.001** | **-8.90 (-17.8 to -0.01); p=.050** | **-58.5 (-91.4 to -25.5); p<0.001** | **-77.2 (-113.0 to -41.5); p<.001** | **-34.1 (-50.2 to -18.0); p<.001** | **-14.4 (-26.9 to -2.05); p=.022** |
| **Water intake (ml/day)** | -0.02 (-0.18 to 0.15); p=.843 | 0.01 (-0.07 to 0.09); p=.796 | -0.20 (-0.48 to 0.08); p=.161 | -0.21 (-0.53 to 0.10); p=.183 | -0.02 (-0.17 to 0.13); p=.753 | -0.06 (-0.17 to 0.05); p=.316 |

|  |  |  |  |  |  |
| --- | --- | --- | --- | --- | --- |
|  | **HDL (x1000)** | **LDL (x1000)** | **Hs-CRP (x1000)** | **SBP (x1000)** | **DBP (x1000)** |
|  |  |  |  |  |  |
| **Energy intake (kcal/day)** | -0.09 (-0.27 to 0.09); p=.343 | -0.35 (-0.94 to 0.23); p=.240 | -0.67 (-3.40 to 2.07); p=.633 | 1.75 (-7.39 to 10.9); p=.708 | 0.54 (-6.40 to 7.48); p=.879 |
| **Protein intake (g/day)** | -0.27 (-2.37 to 1.84); p=.804 | -6.32 (-13.09 to 0.46); p=.068 | -5.54 (-37.3 to 26.2); p=.732 | -10.8 (-116.5 to 95.0); p=.842 | **-101.5 (-181.8 to -21.3); p=.013** |
| **Carbohydrate intake** |  |  |  |  |  |
| Complex carbohydrate (g/day) | -0.19 (-1.10 to 0.72); p=.685 | **5.35 (2.44 to 8.26); p<.001** | -3.27 (-17.0 to 10.4); p=.640 | -3.65 (-49.2 to 41.9); p=.875 | -14.3 (-48.9 to 20.2); p=.416 |
| Simple carbohydrate (g/day) | -0.4 (-1.91 to 1.11); p=.608 | -0.03 (-4.89 to 4.83); p=.992 | 19.0 (-3.14 to 41.2); p=.092 | 36.8 (-38.1 to 111.7); p=.336 | -29.7 (-86.6 to 27.1); p=.306 |
| **Lipid intake** |  |  |  |  |  |
| Saturated fat (g/day) | 3.91 (-1.05 to 8.88); p=.123 | **20.6 (4.69 to 36.6); p=.011** | 4.61 (-70.0 to 79.2); p=.904 | 157.4 (-91.2 to 406.0); p=.215 | **274.9 (86.2 to 463.5); p=.004** |
| Monounsaturated fat (g/day) | -2.41 (-7.49 to 2.67); p=.352 | -1.22 (-16.9 to 14.4); p=.878 | 0.86 (-51.3 to 53.1); p=.974 | -120.6 (-322.9 to 553.8); p=.243 | 69.5 (-84.7 to 223.7); p=.377 |
| Polyunsaturated fat (g/day) | -1.19 (-8.04 to 5.65); p=733 | 0.30(-21.7 to 22.3); p=.979 | 34.8 (-69.1 to 137.9); p=.515 | 210.4 (-133.1 to 553.8); p=.230 | 199.2 (-61.2 to 459.9); p=.134 |
| Cholesterol intake (mg/day) | -0.25 (-0.55 to 0.05); p=.106 | -0.76 (-1.72 to 0.20); p=.121 | 3.83 (-0.70 to 8.4); p=.098 | -11.6 (-26.7 to 3.39); p=.129 | -2.72 (-14.1 to 8.69); p=.641 |
| **Fibre intake (g/day)** | 0.13 (-4.21 to 4.47); p=.952 | **-27.9 (-41.8 to -14.0); p<.001** | **-103.1 (-168.4 to -37.8); p=.002** | -**277.8 (-495 to -60.4); p=.012** | 7.55 (-157.4 to 172.3); p=.928 |
| **Water intake (ml/day)** | -0.00 (-0.05 to 0.03); p=.636 | -0.00 (-0.13 to 0.13); p=.970 | 0.48 (-0.11 to 1.08); p=.111 | -0.83 (-2.84 to 1.18); p=.418 | 0.08 (-1.45 to 1.61); p=.920 |
