# Supplementary material for: Dietary Fibres and the Management of Obesity and Metabolic Syndrome: The RESOLVE Study
Source: Nutrients. 2020 Sep 23;12(10):2911. doi: 10.3390/nu12102911 (PMC7650763; doi:10.3390/nu12102911)
Supplement: Supplementary file 1 [file nutrients-12-02911-s001.zip › supplementary materials/2020-09-22_TableS2_EffectOfFibersOnHealthOutcomesDependingOnTimeGroup.docx]

**Table S2.** Effect of fibre intake on health outcomes depending on time and group - Effect size (95% confidence intervals); p-value.

Significant effect sizes are in bold. This table is the detail of Figure 3. HbA1c: glycated haemoglobin ; Homa-IR: Homeostatic Model Assessment of Insulin Resistance, HDL: high-density lipoprotein, LDL: low-density lipoprotein, Hs-CRP: high-sensitivity c-reactive protein. BMI: Body Mass Index. SBP: systolic blood pressure; DBP: diastolic blood pressure. Re, rE and re: see methods for details. D: day; M: month.

|  |  |  |  |  |  |  |
| --- | --- | --- | --- | --- | --- | --- |
|  | **Central fat** | **Weight (x1000)** | **BMI (x1000)** | **Fat mass** | **Fat free mas** | **Waist (x1000)** |
|  |  |  |  |  |  |  |
| **Re** |  |  |  |  |  |  |
| D20 | -15.0 (-45.9 to 16.0); p=.343 | -0.07 (-0.48 to 0.29); p=.642 | -0.01 (-0.16 to 0.13); p=.847 | -43.7 (-310.7 to 341.2); p=.927 | -95.5 (-253.6 to 62.6); p=.236 | -0.21 (-0.57 to 0.15); p=.252 |
| M3 | -13.7 (-46.4 to 19.0); p=.412 | -0.05 (-0.45 to 0.35); p=.806 | 0.02 (-0.13 to 0.17); p=.793 | -130.6 (-331.4 to 357.7); p=.940 | -1.90 (-168.8 to 165.0); p=.982 | 0.03 (-0.32 to 0.39); p=.858 |
| M6 | -4.0 (-38.5 to 30.5); p=.820 | -0.05 (-0.38 to 0.49); p=.811 | 0.03 (-0.13 to 0.19); p=.747 | -205.7 (-343.2 to 384.1); p=.912 | -8.78 (-184.8 to 167.3); p=.922 | -0.006 (-0.4 to 0.39); p=.978 |
| M12 | -22.7 (-55.9 to 10.6); p=.181 | 0.05 (-0.59 to 0.24); p=.414 | -0.02 (-0.18 to 0.13); p=.776 | -322.1 (-446.3 to 253.3); p=.589 | -15.3 (-184.5 to 153.9); p=.859 | -0.33 (-0.71 to 0.05); p=.087 |
| **rE** |  |  |  |  |  |  |
| D20 | 3.95 (-27.7 to 35.6); p=.807 | -0.09 (-0.24 to 0.58); p=.414 | -0.01 (-0.15 to 0.14); p=.925 | -2.05 (-288.2 to 284.1); p=.989 | 32.4 (-175.6 to 240.4); p=.760 | -0.11 (-0.45 to 0.23); p=.532 |
| M3 | -19.6 (-49.8 to 10.7); p=.205 | -0.05 (-0.56 to 0.22); p=.404 | -0.06 (-0.20 to 0.08); p=.400 | -145.9 (-419.9 to 128.1); p=.297 | -33.2 (-232.7 to 166.3); p=.744 | -0.13 (-0.46 to 0.19); p=.420 |
| M6 | **-41.2 (-68.2 to -14.2); p=.003** | **-0.39 (-0.74 to -0.04); p=.028** | **-0.12 (-0.25 to -0.001); p=.048** | **-302.9 (-547.6 to -58.2); p=.015** | -83.6 (-261.6 to 94.3); p=.357 | **-0.40 (-0.68 to -0.10); p=.008** |
| M12 | **-61.6 (-95.2 to -28.1); p=.000** | **-0.57 (-1 to -0.13); p=.011** | **-0.20 (-0.35 to -0.05); p=.009** | **-442.3 (-746.1 to -138.4); p=.004** | -156.6 (-377.8 to 64.6); p=.165 | **-0.46 (-0.82 to -0.10); p=.012** |
| **re** |  |  |  |  |  |  |
| D20 | -12.5 (-38.5 to 13.5); p=.346 | 0.17 (-0.31 to 0.3); p=.975 | -0.01 (-0.11 to 0.18); p=.634 | -0.01 (-308.3 to 286.9); p=.944 | 20.6 (-175.0 to 216.2); p=.837 | 0.14 (-0.25 to 0.52); p=.486 |
| M3 | -20.8(-45.8 to 4.3); p=.105 | -0.17 (-0.45 to 0.14); p=.295 | -0.06 (-0.17 to 0.11); p=.641 | -0.06 (-437.3 to 136.5); p=.304 | -36.7 (-225.4 to 152.0); p=.703 | -0.09 (-0.46 to 0.28); p=.631 |
| M6 | -20.6 (-45.5 to 4.3); p=.105 | -0.39 (-0.44 to 0.14); p=.322 | -0.12 (-0.17 to 0.11); p=.650 | -0.12 (-446.2 to 122.9); p=.266 | -2.19 (-89.4 to 158.1); p=.982 | -0.06 (-0.43 to 0.31); p=.739 |
| M12 | **-31.9(-57.4 to -6.4); p=.014** | **-0.57 (-0.66 to -0.06); p=.018** | -0.20 (-0.25 to 0.03); p=.121 | **-0.2 (-609.4 to -25.6); p=.033** | -64.7 (-256.7 to 127.3); p=.509 | -0.20 (-0.58 to 0.17); p=.288 |

|  |  |  |  |  |  |  |
| --- | --- | --- | --- | --- | --- | --- |
|  | **Blood glucose (x1000)** | **HbA1c (x1000)** | **Insulimenia** | **Homa-IR (x1000)** | **Total cholesterol (x1000)** | **Triglycerides (x1000)** |
|  |  |  |  |  |  |  |
| **Re** |  |  |  |  |  |  |
| D20 | 0.01 (-0.07 to 0.09); p=.743 | -0.72 (-40.9 to 39.5); p=.972 | **-153.2 (-296.4 to -10.0); p=.036** | -0.09 (-0.24 to 0.07); p=.267 | -0.04 (-0.12 to 0.03); p=.284 | 9.18 (-41.3 to 59.6); p=.721 |
| M3 | 0.02 (-0.06 to 0.1); p=.593 | 20.2 (-19.9 to 60.3); p=.324 | 93.9 (-50.7 to 238.6); p=.203 | 0.10 (-0.05 to 0.26); p=.188 | -0.07 (-0.15 to 0.01); p=.075 | 5.33 (-45.3 to 55.9); p=.836 |
| M6 | 0.03 (-0.06 to 0.12); p=.503 | 26.9 (-17.7 to 71.4); p=.237 | 15.1 (-146.6 to 176.8); p=.855 | 0.05 (-0.12 to 0.22); p=.560 | -0.03 (-0.12 to 0.05); p=.427 | 15.1 (-41.3 to 71.4); p=.600 |
| M12 | 0.02 (-0.06 to 0.11); p=.613 | 9.19 (-33.1 to 51.5); p=.670 | 4.01 (-151.07 to 159.1); p=.960 | 0.04 (-0.12 to 0.21); p=.600 | **-0.09 (-0.17 to -0.01); p=.029** | -0.18 (-71.2 to 36.1); p=.522 |
| **rE** |  |  |  |  |  |  |
| D20 | -0.04 (-0.1 to 0.01); p=.132 | -2.56 (-26.8 to 21.7); p=.836 | -124.3 (-259.8 to 11.3); p=.072 | -0.12 (-0.26 to 0.02); p=.090 | -0.03 (-0.09 to 0.03); p=.281 | -14.6 (-60.4 to 31.3); p=.533 |
| M3 | 0.01 (-0.04 to 0.07); p=.614 | **-31.5 (-54.8 to -8.3); p=.008** | 40.0 (-88.8 to 168.9); p=.543 | 0.06 (-0.07 to 0.19); p=.381 | -0.04 (-0.09 to 0.02); p=.184 | -2.20 (-45.9 to 41.5); p=.921 |
| M6 | **-0.05 (-0.09 to -0.001); p=.045** | **-33.1 (-53.8 to -12.4); p=.002** | 51.0 (-64.4 to 166.5); p=.386 | 0.03 (-0.09 to 0.15); p=.628 | -0.03 (-0.08 to 0.02); p=.189 | -34.4 (-73.4 to 4.68); p=.085 |
| M12 | **-0.06 (-0.12 to -0.01); p=.034** | **-34.0 (-59.7 to -12.4); p=.009** | -60.6 (-202.0 to 80.8); p=.401 | -0.09 (-0.23 to 0.06); p=.243 | -0.003 (-0.06 to 0.06); p=.928 | -9.04 (-57.3 to 39.3); p=.714 |
| **re** |  |  |  |  |  |  |
| D20 | 0.01 (-0.11 to 0.14); p=.843 | 23.5 (-22.2 to 69.2); p=.313 | 12.9 (-150.9 to 176.7); p=.878 | 0.03 (-0.19 to 0.26); p=.764 | 0.03 (-0.03 to 0.10); p=.333 | 16.9 (-59.6 to 93.3); p=.666 |
| M3 | 0.004 (-0.12 to 0.12); p=.948 | -8.29 (-52.37 to 35.8); p=.712 | 106.6 (-51.7 to 264.8); p=.187 | 0.11 (-0.10 to 0.33); p=.308 | 0.06 (-0.01 to 0.12); p=.110 | 1.54 (-72.3 to 75.4); p=.967 |
| M6 | -0.05 (-0.17 to 0.06); p=.370 | -14.9 (-58.6 to 28.7); p=.501 | 27.7 (-126.8 to 182.1); p=.726 | -0.03 (-0.24 to 0.19); p=.794 | 0.02 (-0.05 to 0.08); p=.592 | 4.65 (-68.1 to 77.4); p=.900 |
| M12 | 0.004 (-0.12 to 0.13); p=.943 | -6.86 (-51.6 to 37.9); p=.764 | 78.0 (-81.8 to 237.8); p=.339 | 0.08 (-0.14 to 0.30); p=.482 | 0.06 (-0.004 to 0.13); p=.064 | 10.7 (-64.2 to 85.5); p=.780 |

|  |  |  |  |  |  |
| --- | --- | --- | --- | --- | --- |
|  | **HDL (x1000)** | **LDL (x1000)** | **Hs-CRP (x1000)** | **SBP (x1000)** | **DBP (x1000)** |
|  |  |  |  |  |  |
| **Re** |  |  |  |  |  |
| D20 | -11.6 (-27.5 to 4.31); p=.153 | -34.0 (-100.7 to 32.6); p=.317 | -0.05 (-0.39 to 0.29); p=.785 | 0.21 (-0.70 to 1.12); p=.648 | 0.18 (-0.60 to 0.97); p=.649 |
| M3 | -11.1 (-26.9 to 4.76); p=.170 | -61.2 (-127.6 to 5.18); p=.071 | -0.10 (-0.45 to 0.25); p=.577 | 0.63 (-0.28 to 1.53); p=.178 | 0.39 (-0.40 to 1.17); p=.336 |
| M6 | 6.25 (-11.3 to 23.8); p=.486 | -47.1 (-120.8 to 26.7); p=.211 | -0.01 (-0.40 to 0.37); p=.942 | -0.18 (-1.20 to 0.83); p=.724 | -0.22 (-1.09 to 0.66); p=.629 |
| M12 | 1.60 (-15.1 to 18.3); p=.851 | **-82.0 (-152.1 to -12.0); p=.022** | 0.04 (-0.33 to 0.40); p=.848 | 0.47 (-0.5 to 1.43); p=.343 | -0.26 (-1.09 to 0.57); p=.543 |
| **rE** |  |  |  |  |  |
| D20 | -2.39 (-16.4 to 11.6); p=.737 | -21.7 (-74.2 to 30.8); p=.418 | **0.21 (0.004 to 0.42); p=.046** | 0.08 (-0.78 to 0.93); p=.862 | 0.07 (-0.61 to 0.75); p=.835 |
| M3 | -7.06 (-20.4 to 6.30); p=.300 | -25.8 (-75.9 to 24.2); p=.312 | -0.03 (-0.22 to 0.17); p=.798 | 0.51 (-0.3 to 1.32); p=.217 | 0.41 (-0.24 to 1.06); p=.222 |
| M6 | **13.4 (1.43 to 25.3); p=.028** | -36.6 (-81.3 to 8.05); p=.108 | -0.10 (-0.27 to 0.08); p=.288 | 0.24 (-0.49 to 0.96); p=.519 | 0.23 (-0.35 to 0.82); p=.429 |
| M12 | **17.7 (3.00 to 32.5); p=.018** | -15.0 (-70.3 to 40.3); p=.595 | -0.14 (-0.35 to 0.08); p=.220 | 0.48 (-0.42 to 1.37); p=.298 | 0.20 (-0.52 to 0.92); p=.591 |
| **re** |  |  |  |  |  |
| D20 | 14.3 (-7.68 to 36.3); p=.202 | 8.88 (-46.5 to 64.3); p=.753 | 0.10 (-0.22 to 0.42); p=.540 | -0.13 (-1.27 to 1.02); p=.830 | -0.40 (-1.20 to 0.40); p=.331 |
| M3 | 11.0 (-10.2 to 32.2); p=.309 | 43.6 (-9.87 to 97.1); p=.110 | 0.03 (-0.29 to 0.34); p=.874 | -0.15 (-1.26 to 0.95); p=.787 | -0.07 (-0.84 to 0.71); p=.868 |
| M6 | 10.5 (-10.46 to 31.5); p=.326 | -2.24 (-55.15 to 50.7); p=.934 | -0.06 (-0.37 to 0.25); p=.694 | 0.41 (-0.68 to 1.50); p=.465 | 0.17 (-0.60 to 0.93); p=.666 |
| M12 | 15.3 (-6.20 to 36.8); p=.163 | 47.9 (-6.31 to 102.3); p=.083 | 0.01 (-0.31 to 0.33); p=.945 | 0.75 (-0.37 to 1.87); p=.190 | 0.16 (-0.62 to 0.94); p=.689 |
